# The Effects of Nicardipine on Neuroinflammation and Cognitive Function in Aged Rats Following Abdominal Surgery

**DOI:** 10.3390/jcm14248912

**Published:** 2025-12-17

**Authors:** Nazan Kocaoglu, Hafize Fisun Demir, Fatih Ugun, Elif Aksoz, Bulent Atik, Murat Bıcakcıoglu, Ozlem Sagir, Ahmet Koroglu

**Affiliations:** 1 Department of Anesthesiology and Reanimation, Faculty of Medicine, Balıkesir University, 10145 Balikesir, Turkey; fusdemir@yahoo.com (H.F.D.); drfugun@yahoo.com (F.U.); bulent_atik@yahoo.com (B.A.); ozlemsagir@yahoo.com (O.S.); koroglua@yahoo.com (A.K.); 2 Department of Medical Pharmacology, Faculty of Medicine, Balikesir University, 10145 Balikesir, Turkey; elifaksoz@yahoo.com; 3 Department of Anesthesiology and Reanimation, Faculty of Medicine, Inonu University, 44280 Malatya, Turkey; drmuratft@gmail.com

**Keywords:** anaesthesia, neuroinflammation, nicardipine, POCD, surgery

## Abstract

**Background/Objectives**: Postoperative cognitive dysfunction (POCD) is a serious complication of anaesthesia/surgery. The present study investigated the effects of nicardipine—a calcium channel blocker—on neuroinflammation and POCD in rats. **Methods**: Following ethical approval, 30 Wistar albino rats were divided into three groups: control (Group C), surgery (Group S), and surgery and nicardipine (a single intraperitoneal dose of 5 mg/kg nicardipine) (Group N). Cognitive function was assessed 48 h postoperatively using the MWM test. The rats were sacrificed on the 5th day, and hippocampi were isolated and frozen at −80 °C on the same d ay. Hippocampal tissues were homogenised; ELISA and Western blot tests were performed to assess IL-1, IL-6, TNF-α, and caspase-3. **Results**: All groups showed a significant decrease in the time required to locate the hidden platform from day 1 to day 4. In the probe trial of the Morris water maze test, Group C spent more time in the target quadrant compared with Group S, indicating surgery-related cognitive impairment. The ELISA and Western blot analyses demonstrated that the hippocampal levels of IL-1, IL-6, TNF-α, and caspase-3 were significantly elevated in both Groups S and N compared with the controls. No statistically significant differences were observed between Groups S and N, indicating that the measured cognitive performance and hippocampal inflammatory responses were comparable between these groups. **Conclusions**: This study showed that a single intraperitoneal dose of 5 mg/kg of nicardipine did not measurably improve early postoperative cognitive performance or reduce hippocampal inflammation. In particular, nicardipine did not have a detectable effect on early postoperative neuroinflammation or cognition at the tested dose and timing in this rat model. Further studies exploring different doses and timing or repeated administration would help to clarify its potential role.

## 1. Introduction

Postoperative cognitive dysfunction (POCD) is a serious central nervous system complication, characterised by memory loss, impaired intellectual abilities, and anxiety, often occurring in elderly individuals following surgery and anaesthesia [1,2]. In patients over the age of 65, the incidence of POCD occurring within the first seven days after surgery ranges from 9% to 54% [3]. The patient’s educational level, preoperative cognitive function, and existing comorbidities have been identified as significant contributors to the development of POCD. Additionally, factors related to the management of surgery and anaesthesia may affect the development of POCD [4].

Current hypotheses on the pathogenesis of POCD are related to mechanisms such as central nervous system inflammation, cholinergic system dysfunction, oxidative stress, apoptosis, imbalances of neurotransmitter levels, and changes in neuronal synaptic plasticity [5,6,7]. Surgical stress activates the immune system, resulting in the release of inflammatory signalling molecules both locally and systemically. The role of anaesthesia in the development of POCD remains to be fully elucidated. It is hypothesised that the direct toxic effects on calcium homeostasis in neurons, the inhibition of the physiological functions of neuronal stem cells, and the acceleration of neurodegenerative processes contribute to this process [8].

Calcium ions regulate vital physiological processes such as synaptic plasticity, excitatory amino acid transmission, and neuronal apoptosis. Disruption of mitochondrial calcium homeostasis, caused by anaesthesia and surgery, can lead to abnormalities in neuronal synaptic connections [6,9]. Recent clinical and experimental studies have investigated the effects of calcium channel blockers on cognitive function [10,11,12,13]. Nicardipine, a dihydropyridine derivative, is a voltage-sensitive calcium channel blocker used to treat vascular disorders. While it reflects the general properties of dihydropyridines, it is specifically used to treat hypertension following acute traumatic brain injury, ischaemic stroke, and subarachnoid haemorrhage due to its high selectivity for cerebral and coronary arteries. In addition to its effects on calcium signalling in glial cells, there is evidence that it inhibits the release of TNF-α and IL-6 by inhibiting microglial cell activation [1,2]. Although nimodipine and other calcium channel blockers have been investigated extensively for their effects on cognitive function, research on nicardipine and postoperative cognitive dysfunction is limited.

Based on the above findings, this study aimed to assess the role of nicardipine hydrochloride in modulating neuroinflammation and postoperative cognitive dysfunction in aged rats subjected to abdominal surgery under general anaesthesia.

## 2. Materials and Methods

### 2.1. Ethics Statement

This study was approved by the Local Animal Experiments Ethics Committee (Decision No. 2021/3−7), and all procedures were performed following the Guide for the Care and Use of Laboratory Animals issued by the National Institutes of Health.

### 2.2. Experimental Animals

Thirty male Wistar albino rats (20–21 months old, 550–750 g) were included. They were supplied by the Local Laboratory Animal Production, Care, Application, and Research Centre. During the experiment, the rats were given free access to food and water and housed in a room with a 12-h light/12−h dark cycle. The temperature was kept at 21 ± 3 °C and the humidity at 55% ± 5%. The rats were transferred to the intervention room at least 24 h before the behavioural experiments so that they had time to adapt. All behavioural tests were completed between 8 am and 12 am.

### 2.3. Drugs and Experimental Protocol

**Figure 1:**
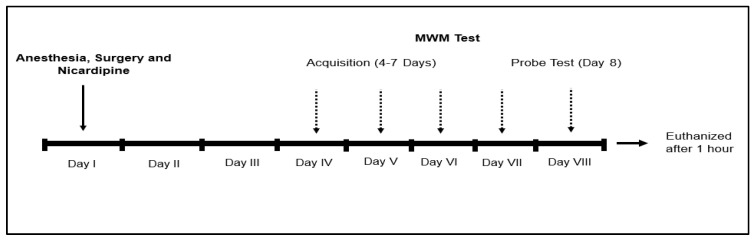
The experimental protocol.

Thirty male aged rats (10 rats in each group) were randomly divided into three groups:

Control (C) group (*n* = 10)—cognitive test only, no anaesthesia or surgery;

Surgery (S) group (*n* = 10)—laparotomy under isoflurane anaesthesia;

Nicardipine (N) group (*n* = 10)—laparotomy under isoflurane anaesthesia followed by intraperitoneal (i.p.) administration of 5 mg/kg nicardipine hydrochloride (Nicardipine Aguettant 10 mg/10 mL, Polifarma, Istanbul, Turkey) (Figure 1).

The sample sizes per group for analyses were as follows:

Behavioural/cognitive tests—*n* = 10 animals per group;

Biochemical analyses (ELISA and Western blot)—*n* = 6 animals per group.

All animals were included in the behavioural assessments. For biochemical analyses, 6 animals per group were used for each endpoint.

### 2.4. Anaesthesia and Surgery

The rats were prepared in an anaesthesia box, and their abdominal areas were shaved. Then, 2% isoflurane was administered via inhalation using an anaesthesia machine (Plexx, HNG 6, Elst, The Netherlands) in an oxygen–air (1:1) mixture. The abdominal region was cleaned with iodine, and a 3 cm vertical incision was made along the midline for a laparotomy. The small intestine was then removed from the abdomen, gently massaged for three minutes, and replaced in the intra-abdominal cavity. This procedure was repeated every 10 min for one hour. Anaesthesia was maintained with 1.5–2% isoflurane. At the end of the operation, the nicardipine group received 5 mg/kg of nicardipine (Nicardipine Aguettant 10 mg/10 mL, Polifarma, Istanbul, Turkey) intraperitoneally. Analgesia was provided by administering 0.003 mg/kg of bupivacaine (Buvicaine 0.5%, Polifarma, Istanbul, Turkey) through the incision line. Haemostasis was achieved, the abdomen was closed with sterile sutures, and the volatile agent was stopped. Each rat was caged separately and monitored until conscious.

### 2.5. Morris Water Maze (MWM) Test

Cognitive function was assessed 48 h after surgery using the Morris water maze test. The test was conducted in a circular tank (150 cm in diameter and 50 cm in depth), which was filled with water to a depth of approximately 30 cm. Non-toxic black tempera paint was added to make the water opaque. The water temperature was set to 25 ± 1 °C. The tank was divided into four equal quadrants (north, south, east, and west). A plexiglass platform with a diameter of 12 cm was placed 1 cm below the water level at a point close to the centre of one of the quadrants (in this study, the southeast quadrant was used). The test was conducted over four consecutive days (the acquisition period), with each rat swimming three times a day. The order and direction in which the rats were released into the tank were predetermined for the first four days. During each swimming session, the rats were given 60 s to locate the platform. Those that did not find the platform within this time were placed on it by the researcher for 20 s. The time taken by each rat to find the platform was recorded after every test. On day 5 (the probe test), the platform was removed and the rats swam for a single 60-s session. The time that each rat spent in the former platform area was recorded using a digital video camera. These recordings were analysed using the Ethovision XT software (Version 10, Noldus Information Technologies, Wageningen, The Netherlands).

### 2.6. Sample Collection

One hour after the probe test, rats were anaesthetised with ketamine–xylazine (Ketamine hydrochloride (Ketalar, 100 mg/10 mL, Pfizer Pharmaceuticals Ltd., Istanbul, Turkey) and the sedative xylazine hydrochloride (Rompun, 2% 25 mL, Bayer Turk Chemical Industry, Inc., Istanbul, Turkey) and sacrificed. Whole brains were removed, and hippocampi were separated and stored at −80 °C.

### 2.7. Biochemical Analyses

Hippocampal samples were homogenised and centrifuged, and their supernatants were separated. ELISA and Western Blot analyses were performed to assess proinflammatory markers (IL-1β, IL-6, TNF-α) and apoptosis (caspase-3) (Atlas Biotechnology Laboratory, Ankara, Turkey).

#### 2.7.1. ELISA Protocol

Hippocampal tissues were homogenised on ice in saline to measure protein concentrations. IL-1β (Cat. No.: E0119Ra), IL-6 (Cat. No.: E0135Ra), TNF-α (Cat. No.: E0764Ra), and caspase-3 (Cat. No.: E1648Ra) levels were assessed using an ELISA kit (Bioassay Technology Laboratory, Shanghai, China), following the manufacturer’s instructions.

#### 2.7.2. Western Blot Protocol

The protein concentration in the hippocampal tissue was measured with a Bradford Protein Assay Kit (ABP Biosciences, Rockville, MD, USA). Twenty micrograms of protein obtained from all cell lysates was separated via SDS-PAGE and transferred to a nitrocellulose membrane using the Iblot system. Membranes were blocked with 5% BSA in PBS-T, incubated overnight at 4 °C with a primary antibody, and washed three times in PBS-T. Afterward, they were incubated for 1 h at room temperature with a 1:10,000-diluted HRP-conjugated secondary antibody in blocking solution, followed by three additional PBS-T washes. Signals were detected using an enhanced chemiluminescence substrate (NZY Supreme ECL HRP Substrate, Nzytech, Lisbon, Portugal, Cat. No.: Mb19301), and images were captured on an imaging device (ChemiDoc-It2, UVP, Upland, CA, USA). Following the Western blot experiment, ImageJ (version 1.53) software was used to analyse the results via densitometry, which involves converting the light intensity of the protein bands into numerical values. The darker or brighter the band, the greater the amount of protein present. The ImageJ software was used to measure the pixel density of each band, yielding a value known as the integrated density. The results were then normalised using a loading control, such as ACTB or GAPDH, and the band image displayed on the device was converted into comparable numerical protein quantities.

### 2.8. Statistical Analysis

Prism 6.0 software (GraphPad Software, Inc., San Diego, CA, USA) was used for statistical data analysis. Results were expressed as the mean ± SEM. The acquisition period (1–4 days) of the MWM was analysed with a two-way analysis of variance (ANOVA), followed by Bonferroni post hoc tests. Other results were evaluated via one-way ANOVA followed by Bonferroni post hoc tests. Results were considered statistically significant if *p* < 0.05.

## 3. Results

### 3.1. Effects of Nicardipine on MWM Test Outcomes

**Figure 2:**
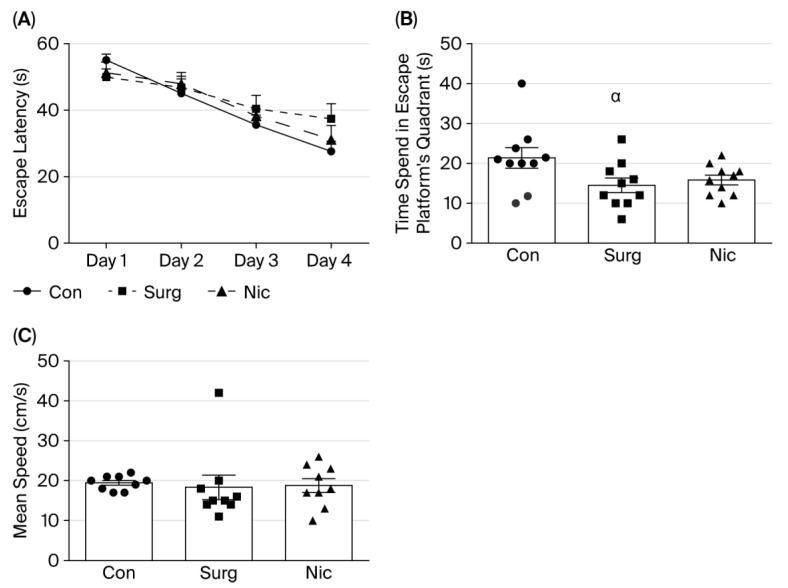
(**A**) Results of control, surgery, and nicardipine groups in the acquisition trial of the MWM test. Each value represents the mean ± SEM. (**B**) Results of control, surgery, and nicardipine groups in the probe trial of the MWM test. Each value represents the mean ± SEM (*n* = 10); ^α^ *p* < 0.05 compared with the control group. (**C**) Mean swimming speeds of rats in the three experimental groups as measured in the MWM test. There were no statistically significant differences between the groups. Con: control; Surg: surgery; Nic: nicardipine.

In all three groups, the time taken to find the hidden platform was shortened significantly from day 1 to day 4 [two-way ANOVA, effect of day F (3,36) = 22.28, *p* < 0.05] (Figure 2A). Tukey’s multiple comparison test showed that the latency time decreased significantly on each successive day of the acquisition test (*p* < 0.05) (Figure 2A).

In the probe trial of the MWM test, rats in Group C spent significantly more time in the quadrant containing the escape platform compared to Group S [one-way ANOVA, F (2,27) = 3.478, *p* < 0.05] (Figure 2B). However, post hoc comparisons did not reveal any statistically significant differences between Group N and the other two groups (Figure 2B). Swimming speeds were similar in all three groups (Figure 2C).

### 3.2. Effects of Nicardipine on IL-1β, IL-6, TNF-α, and Caspase-3 Levels in the Hippocampus

To evaluate the effects of nicardipine on cognitive function, the concentrations of hippocampal IL-1β, IL-6, TNF-α, and caspase-3 were measured in six samples from each group.

#### 3.2.1. ELISA Analysis

**Figure 3:**
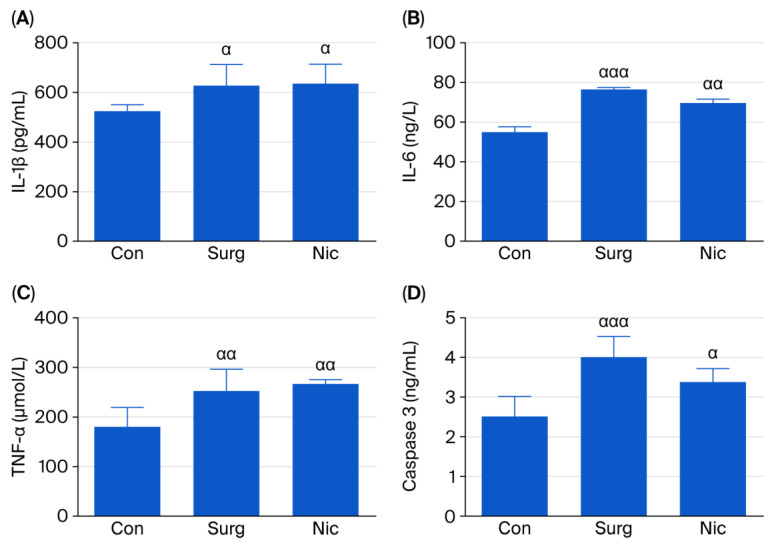
(**A**) The levels of IL-1β in the hippocampi of the control, surgery, and nicardipine groups. Each value represents the mean ± SEM (*n* = 6); ^α^ *p* < 0.05 compared with the control group. (**B**) The levels of IL-6 in the hippocampi of the control, surgery, and nicardipine groups. Each value represents the mean ± SEM (*n* = 6); ^ααα^ *p* < 0.001, ^αα^ *p* < 0.01 compared with the control group. (**C**) The levels of TNF-α in the hippocampi of the control, surgery, and nicardipine groups. Each value represents the mean ± SEM (*n* = 6); ^αα^ *p* < 0.01 compared with the control group. (**D**) The levels of caspase-3 in the hippocampi of the control, surgery, and nicardipine groups. Each value represents the mean ± SEM (*n* = 6); ^ααα^ *p* < 0.001, ^α^ *p* < 0.05 compared with the control group. This figure shows the distribution of mediators as analysed via ELISA.

The IL-1β, IL-6, and TNF-α levels were significantly higher in Groups S and N compared to the control group [one-way ANOVA; F (2,15) = 4.990, *p* < 0.05; F (2,15) = 30.99, *p* < 0.05; F (2,15) = 10.91, *p* < 0.05, respectively]. However, the results for all three mediators were similar between Groups S and Group N (Figure 3A–C). The caspase-3 levels were also significantly higher in Groups S and N compared to the control group [one-way ANOVA, F (2,15) = 15.99, *p* < 0.05], but no statistically significant difference was detected between these two groups (Figure 3D).

#### 3.2.2. Western Blot Analysis

**Figure 4:**
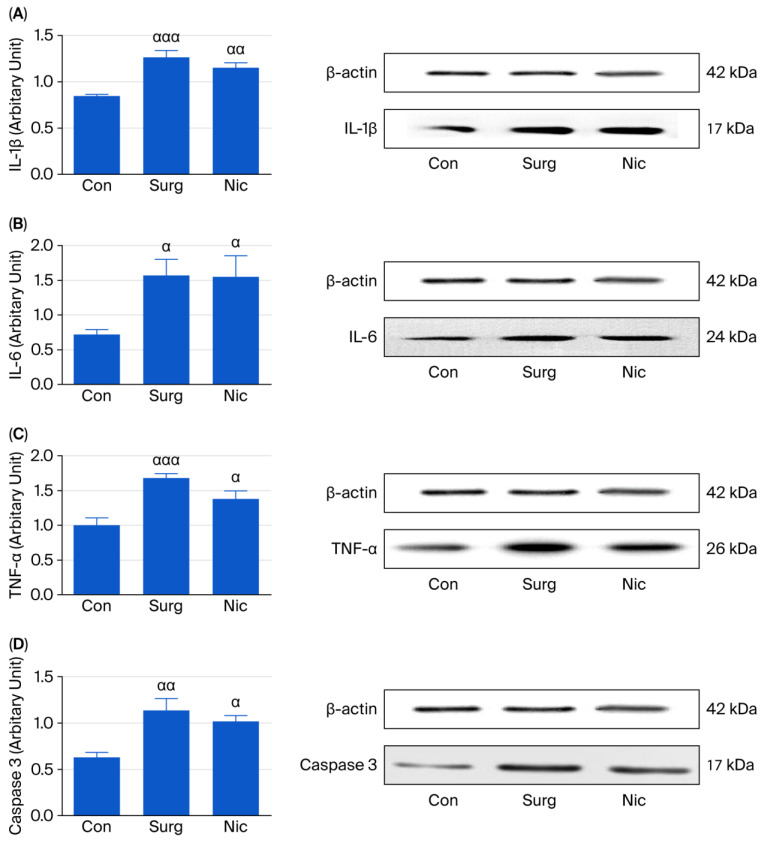
(**A**) The levels of IL-1β in the hippocampi of the control, surgery, and nicardipine groups. Each value represents the mean ± SEM (*n* = 6); ^ααα^ *p* < 0.001, ^αα^ *p* < 0.01 compared with the control group. (**B**) The levels of IL-6 in the hippocampi of the control, surgery, and nicardipine groups. Each value represents the mean ± SEM (*n* = 6); ^α^ *p* < 0.05 compared with the control group. (**C**) The levels of TNF-α in the hippocampi of the control, surgery, and nicardipine groups. Each value represents the mean ± SEM (*n* = 6); ^ααα^ *p* < 0.001, ^α^ *p* < 0.05 compared with the control group. (**D**) The levels of caspase-3 in the hippocampi of the control, surgery, and nicardipine groups. Each value represents the mean ± SEM (*n* = 6); ^αα^ *p* < 0.01, ^α^ *p* < 0.05 compared with the control group. Con: control, Surg: surgery, Nic: Nicardipine. This figure shows the distribution of mediators as analysed via Western blot.

The IL-1β, IL-6, and TNF-α levels were significantly increased in Groups S and N compared to the control group [one-way ANOVA; F (2,15) = 15.92, *p* < 0.05; F (2,15) = 4.65, *p* < 0.05; F (2,15) = 11.66, *p* < 0.05, respectively]. However, no statistically significant differences were found between Groups S and N (Figure 4A–C). Furthermore, a significant increase in caspase-3 was seen in both Groups S and N compared to the control group [F (2,15) = 8.90, *p* < 0.05]. No statistically significant difference was found between the two groups (Figure 4D).

## 4. Discussion

This study evaluated the effects of nicardipine (5 mg/kg) administered intraperitoneally on cognitive function and the hippocampal inflammatory response in aged rats. The MWM test showed that the cognitive decline caused by the surgical intervention was not improved significantly with nicardipine administration. Furthermore, an analysis of inflammatory markers in the hippocampus indicated that nicardipine did not significantly reduce neuroinflammation.

The molecular basis for changes in calcium (Ca^2+^) metabolism in the brain associated with ageing has not yet been elucidated; however, dysregulation of homeostasis is frequently linked to cognitive decline. Uryash et al. [14] demonstrated that the process of ageing was associated with a significant increase in neuronal Ca^2+^ levels. Furthermore, they showed that the cognitive impairment that develops after anaesthesia is closely related to intracellular Ca^2+^ accumulation. L-type calcium channels (LTCCs) play a role in regulating synaptic plasticity by increasing intracellular free Ca^2+^ levels. The increased expression of these channels with age leads to Ca^2+^ accumulation in hippocampal neurons and impaired synaptic plasticity [12]. Inflammatory cytokines affect neuronal activity through increased intracellular calcium. Prior research has identified that high TNF-α levels increase calcium release from intracellular stores and enhance LTCC function in cultured neurons [12]. In recent years, numerous studies on LTCC inhibition and its effects on cognitive function have focused specifically on nimodipine.

In the study by Zhang et al. [15], a POCD model based on aged rats using sevoflurane and surgery was examined. The findings revealed that the administration of 1 mg/kg i.p. nimodipine prior to laparotomy led to a reduction in hippocampal Ca^2+^ accumulation and calcineurin-mediated neuronal apoptosis. This, in turn, resulted in a significant improvement in learning and memory performance. A previous study showed that administering nimodipine at a dose of 1 mg/kg intraperitoneally (i.p.) prior to splenectomy suppressed intracellular Ca^2+^ and increased Bax/Bcl-2, consequently preserving cognitive function [9].

In another work, Yang et al. [16] reported that a three-month dietary treatment with nimodipine in rats with cerebral small-vessel disease decreased the risk of stroke and preserved vascular and cognitive function. Furthermore, a study demonstrated that cognitive impairment resulting from cerebral ischaemia was significantly improved by nimodipine treatment, which prevented excessive Ca^2+^ influx into mitochondria [17]. Haile et al. [18] also reported a significant improvement in cognitive function after moderate hypoxia in adult rats given nimodipine. In another study, it was shown that the NF-κB, TNF-α, and IL-1β levels in the hippocampus were significantly increased in rats with vascular dementia that were not treated with nimodipine. Treatment with nimodipine resulted in a reduction in inflammation by suppressing this response [19]. While these studies provide information about the relationship between LTCC blockade and cognitive function, the existing literature on other dihydropyridine derivatives is more limited. Nicardipine is a dihydropyridine-derived LTCC antagonist. In a study investigating the protective effects of hypotension induced by the i.p. administration of nicardipine (40 mg/kg), nimodipine (40 mg/kg), and nitroglycerin (30 mg/kg) on short- and long-term memory in mice following a cognitive performance test, the protective effect of nicardipine on long-term memory was shown to be weaker than that of nimodipine [20]. The anti-neuroinflammatory effects of nicardipine on microglial cells were also investigated in experimental research. It was established that the intraperitoneal administration of nicardipine or saline to mice over a period of three days resulted in reduced lipopolysaccharide-induced increases in IL-6 and TNF-α—and the associated microglial activation—in the group receiving a daily dose of 5 mg/kg nicardipine [21].

Although experimental models have demonstrated that LTCC antagonists improve cognitive abilities, clinical studies have yielded conflicting results [12]. Nimodipine treatment has been reported to improve cognitive and behavioural symptoms in Alzheimer’s patients [10]. A Cochrane review published in 2002 evaluated 14 randomised controlled trials involving 3166 patients. This review reported that nimodipine treatment may provide a certain level of benefit in patients diagnosed with Alzheimer’s disease, dementia due to cerebrovascular events, and mixed-type dementia. However, it should be noted that the role of voltage-dependent calcium channels in the pathogenesis of vascular dementia is limited. Therefore, calcium channel antagonists such as nimodipine may have minimal therapeutic efficacy in such dementias [22]. The results of another multicentre randomised controlled trial indicated that patients receiving 30 mg of nimodipine three times daily for six months following acute ischaemic stroke displayed no significant cognitive decline in comparison to those administered a placebo. However, a marked improvement was observed in long-term memory assessments [23]. In a different study of elderly patients undergoing radiofrequency ablation for atrial fibrillation, it was found that nicardipine alone or in combination with esmolol reduced the incidence of POCD [24]. In patients undergoing orthognathic surgery, controlled hypotension with nitroglycerin and nicardipine has been demonstrated to have no effect on cerebral oxygen saturation and postoperative cognitive function [25].

The present study suggests that the failure of nicardipine administration to significantly reduce surgery- and anaesthesia-induced cognitive impairment and the hippocampal proinflammatory response in aged rats may have been related to the pharmacokinetic and pharmacodynamic properties of the drug. While both nicardipine and nimodipine are classified as dihydropyridine calcium channel blockers, they differ in terms of central nervous system penetration and lipophilic properties. The literature indicates that nimodipine is capable of crossing the blood–brain barrier due to its highly lipophilic structure, reaching significant concentrations in the brain tissue and cerebrospinal fluid [26]. Zheng et al. [27] hypothesised that, in addition to blocking L-type calcium channels, nimodipine may exert effects through novel molecular targets such as monoamine oxidase A (MAOA). They further posited that this may result in a beneficial effect in the treatment of neurodegenerative diseases through oxidative stress modulation. Sadan et al. [28] reported that the plasma concentrations measured in patients with a subarachnoid haemorrhage who received intrathecal nicardipine were lower than the agent concentration in the cerebrospinal fluid. Considering the data reported in the extant literature regarding the higher central nervous system penetration of nimodipine, the absence of a significant improvement in cognitive impairment and hippocampal inflammation in the present study may be related to the distribution of the drug within the central nervous system. In addition to the pharmacokinetic properties of nicardipine, the timing of administration and the treatment dose should be considered as other factors that may have influenced the results of this study. In some experimental models, nimodipine has been administered in a single dose in the preoperative period [9,15]. Another study evaluated the effects of long-term nimodipine treatment on cognitive function [16]. Haile et al. [20] reported that a single dose of 40 mg/kg i.p. nicardipine was not as effective as nimodipine regarding long-term memory. Another study reported that repeated doses of 5 mg/kg i.p. nicardipine suppressed neuroinflammation [21]. In the present study, a single intraoperative dose of 5 mg/kg nicardipine was administered intraperitoneally. The variability in the results between studies can be explained by the heterogeneity of the experimental models employed and the range of treatment doses administered.

The present study has several limitations. First, the dosage of nicardipine administered was 5 mg/kg, but it is acknowledged that different doses may be more efficacious in altering the effects of the drug on cognitive impairment and neuroinflammation. Furthermore, cognitive decline was evaluated exclusively in the short term, with no consideration of long-term effects.

## 5. Conclusions

The administration of nicardipine following anaesthesia and surgery did not demonstrate a significant protective effect against cognitive impairment and or the hippocampal inflammatory response. In future studies, it is important to evaluate potential drug combinations using different administration protocols and dosage regimens to clarify the possible neuroprotective effects of nicardipine against POCD. Moreover, studies that compare nicardipine with other calcium channel blockers will contribute to a more complete understanding of the role of this drug group in clinical practice.

## Data Availability

Data are contained within the article.

## Abbreviations

The following abbreviations are used in this manuscript:

POCD	Postoperative Cognitive Dysfunction
MWM	Morris Water Maze Test
LTCC	L-Type Calcium Channel
i.p.	Intraperitoneally
MAOA	Monoamine Oxidase A
